# Laparoscopic drainage of an intramural duodenal haematoma: a novel technique and review of the literature

**DOI:** 10.1186/1749-7922-6-42

**Published:** 2011-12-20

**Authors:** Gregory J Nolan, Cino Bendinelli, Jon Gani

**Affiliations:** 1 Division of Surgery, University of Newcastle, John Hunter Hospital, New Lambton Heights, NSW, 2310, Australia

## Abstract

Intramural Duodenal Haematoma (IDH) is an uncommon complication of blunt abdominal trauma. IDH's are most often treated non-operatively. We describe laparoscopic treatment of an IDH after failed conservative management. To our knowledge, successful laparoscopic drainage of an IDH in an adult has not been described previously in the literature.

## Introduction

Intramural Duodenal Haematoma (IDH) is uncommon and may follow high energy blunt abdominal trauma. It accounts for 2% of injuries in children in this setting [1]. It is also seen in minor abdominal injuries in thrombasthenic patients [2] and endoscopic duodenal procedures [3]. The position of the duodenum over the vertebral column and its attachment to the ligament of Treitz predisposes it to deceleration injuries. Deceleration may cause IDH due to the shearing of mucosa and submucosa which disrupts the submucosal vascular plexus [4]. Historically IDH was managed surgically [4,5]. At laparotomy the surgical options included simple haematoma evacuation, gastroenterostomy with or without pyloric exclusion, duodenoduodenostomy, duodenojejunostomy or rarely pancreatoduodenectomy, depending on the severity of injury [5,6]. The introduction and establishment of Total Parenteral Nutrition (TPN) allowed the shift toward a more conservative approach [6-12]. TPN provides the nutritional requirements while awaiting resolution of the gastric outlet obstruction caused by the IDH. Today, IDH is primarily treated non-operatively and surgery considered only if the gastric outlet obstruction is not resolved in approximately 14 days [7]. Table 1 details surgical and radiological interventions in the literature which have been used for the management of IDH in blunt abdominal trauma. In this report we describe a novel laparoscopic technique for successful drainage of an IDH and review the surgical and radiological interventions reported in the literature.

**Table 1:** Literature review of interventions for Intramural Duodenal Haematomas

Author	Year	N° of Cases	Days to Drainage	Procedure Performed	Outcome
Benieghbal et al [13].	2008	1	9	Laparoscopic drainage and omental patch	Discharged day 3 post-surgery. Normal barium meal at 4 weeks. Asymptomatic at 6 months follow-up.

Hanish and Pappas [12]	2007	1	19	Percutaneous CT guided drainage	Discharged day 1 post-procedure. CT 10 days after discharge showed complete resolution.

Desai et al [15]	2003	2	< 1	Laparotomy and drainage	No duodenal stricture or fistula on follow-up.

Takishima et al [16]	2000	1	6	Laparotomy and evacuation of haematoma	Radiologic resolution on CT on the 40th postoperative day.

Maemura et al [14]	1999	1	4	Laparoscopy converted to open to repair duodenal perforation	Discharged day 16 post-surgery.

Jewett et al [1]	1988	38	< 1	24: evacuation of haematoma 14:bypass procedure*	Mean hospital stay 14.2 days.

Jewett et al [1]	1988	83	> 1	65: evacuation of haematoma 18: bypass procedure*	Mean hospital stay 16 days.

* Bypass procedures: gastroenterostomy, duodenojejunostomy, duodenoduodenotomy

## Case Report

**Figure 1:**
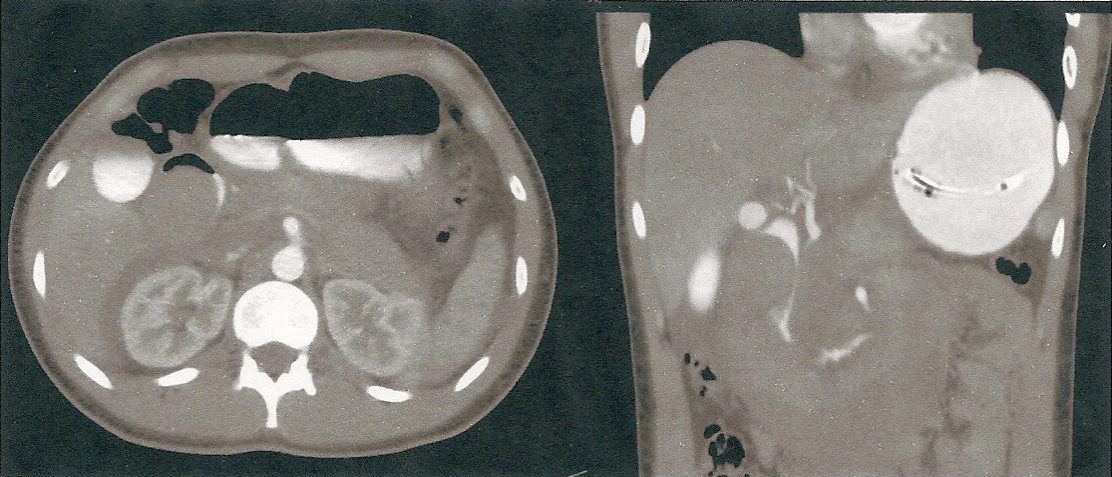
**Axial and coronal view at Computer Tomography with oral and intravenous contrast**. The Intramural Duodenal Haematoma extends from D2 to the duodenal-jejunal junction.

An 18 year old male sustained blunt abdominal trauma after falling off a skateboard onto a tree stump. Three days after the injury, he presented to a peripheral hospital complaining of increasing left upper quadrant abdominal pain. He was transferred to a Level 1 Trauma Centre for further management. On arrival he was afebrile and haemodynamically normal. His abdomen was distended with generalised tenderness and guarding. Pathology revealed a normal full blood count, liver function tests and coagulation studies. The lipase was raised to 2928 U/l (NR < 346). Computer Tomography with pancreatic imaging protocol demonstrated an intramural haematoma extending from D2 to the duodenal-jejunal flexure (Figure 1). There was near complete obstruction of the duodenal lumen associated with a distended D1 and stomach. There were no other significant injuries. A trial of non-operative management with TPN and nasogastric tube (NGT) decompression was instituted.

On day ten a progress CT scan was performed showing no change in size of duodenal haematoma. On day thirteen, the gastric outlet obstruction had not resolved. The risks of surgery including haemorrhage, duodenal leak and fistula formation were weighed against the ongoing conservative approach with an extended period of TPN and the potential for duodenal structuring. The non-operative approached was abandoned.

## Operative Technique

**Figure 2:**
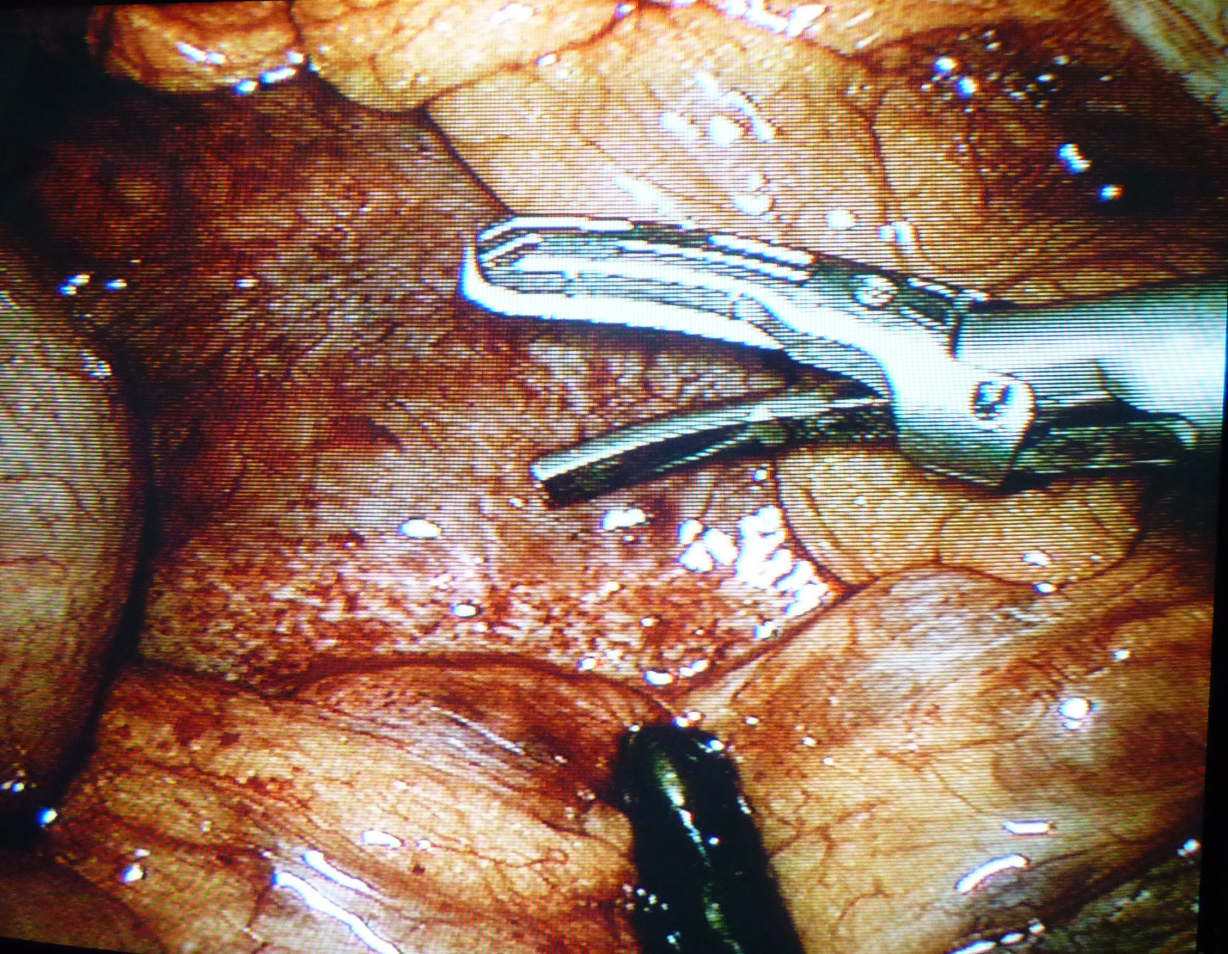
**The inframesocolic portion of the Intramural Duodenal Haematoma before incision with harmonic scalpel**.

**Figure 3:**
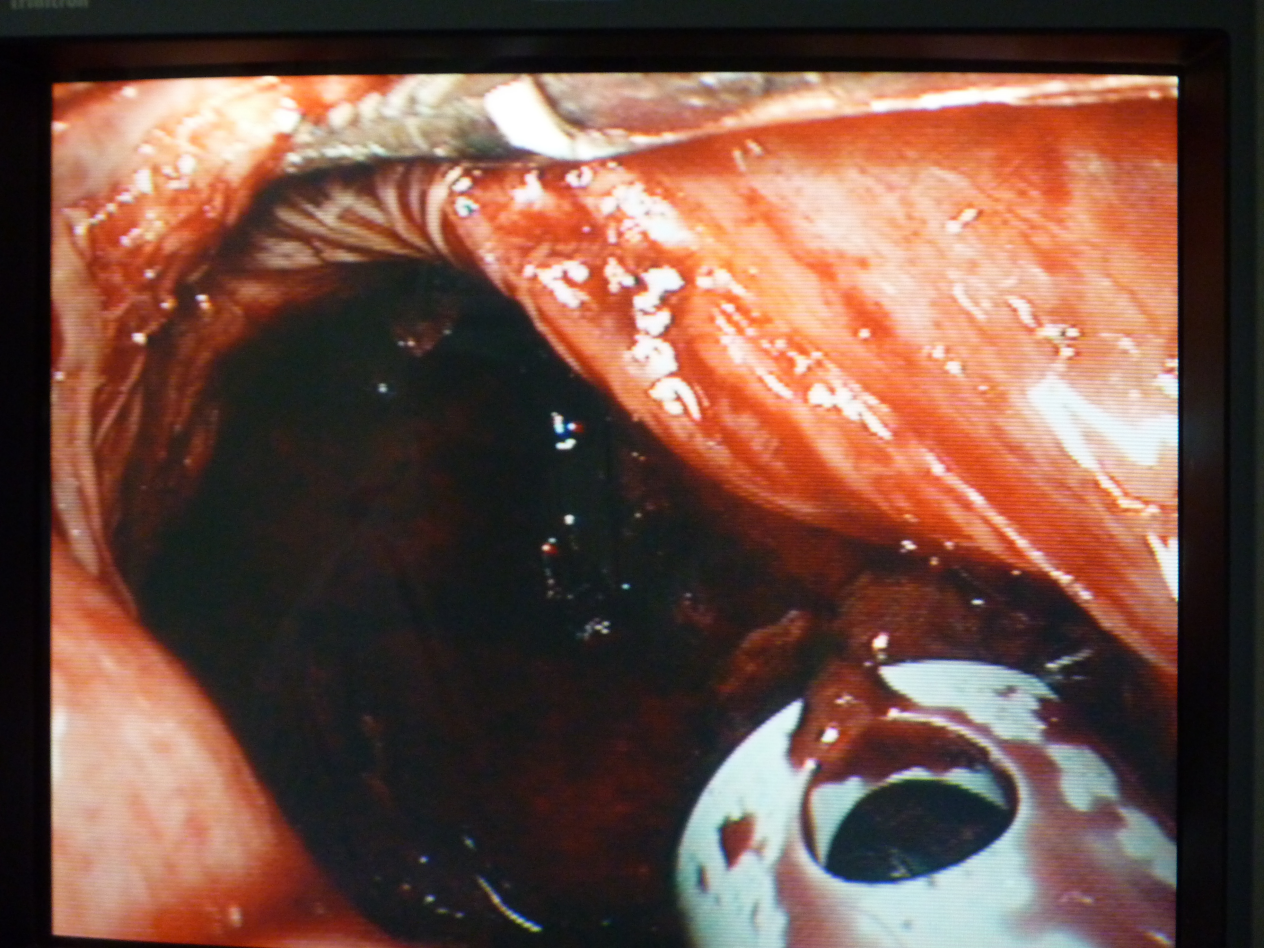
**Intramural Duodenal Haematoma cavity after clot evacuation**.

**Figure 4:**
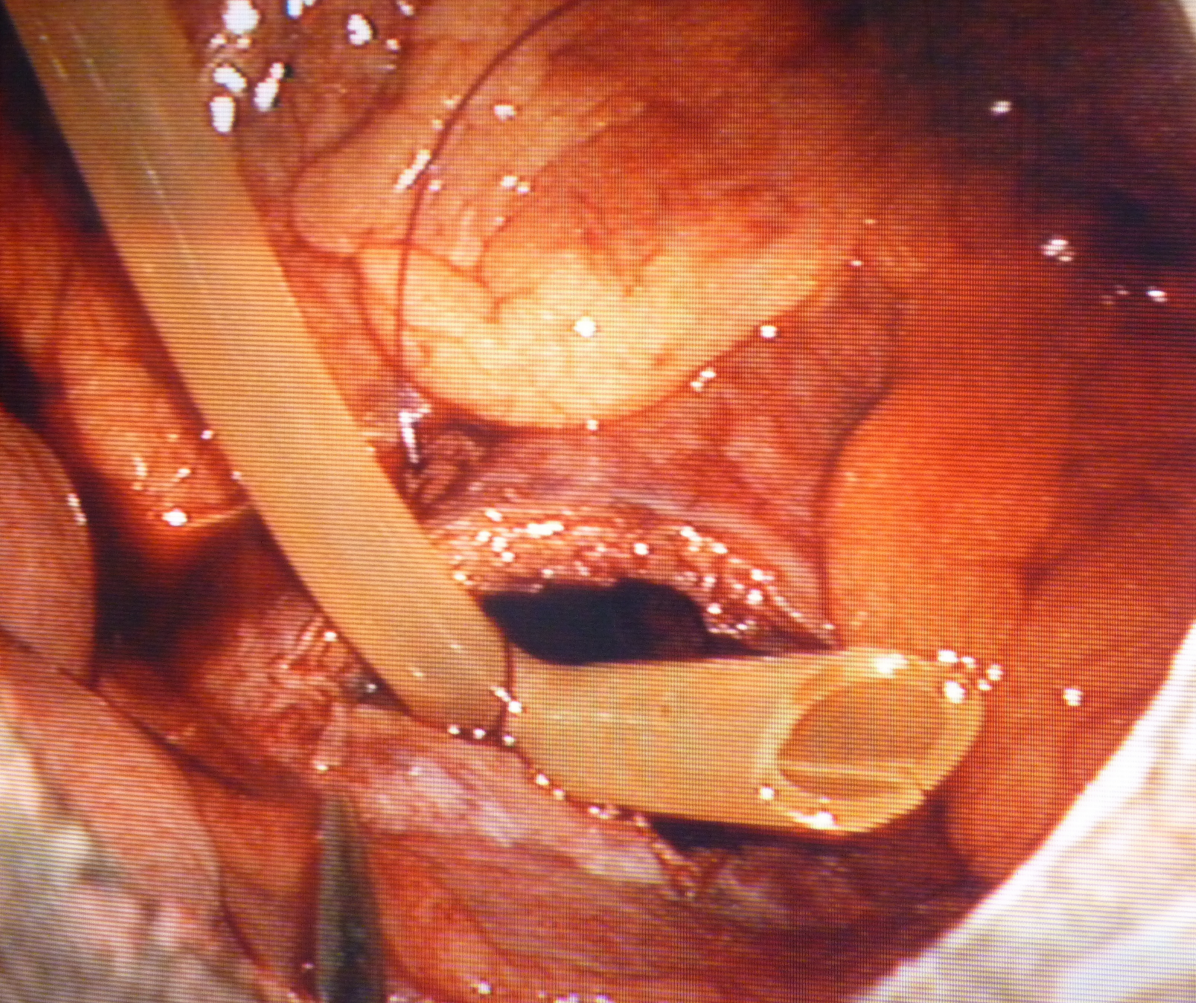
**Insertion of T-tube post evacuation of blood clot**.

**Figure 5:**
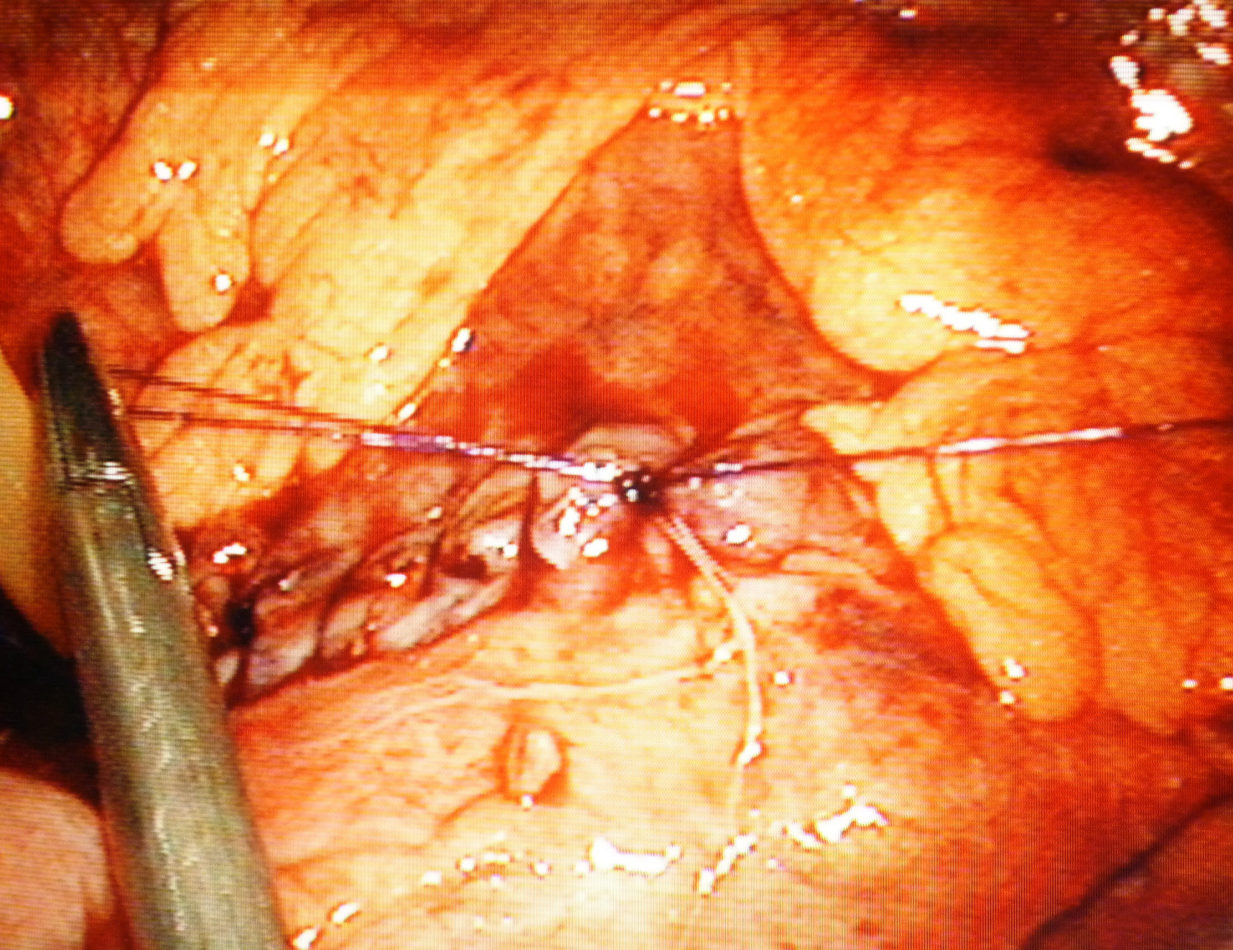
**Seromuscular layer sutured with a 3-0 PDS continuous suture**. Note T-tube seen to the left of the needle holders.

Under general anaesthesia, laparoscopic drainage of the IDH was performed using a 4 port technique. An umbilical Hasson port and two 10 mm ports in the left and right lower quadrants were inserted. One 5 mm port in the right upper quadrant was also inserted. The omentum and transverse colon were elevated and the IDH in the third part of the duodenum (D3) was approached infracolically. No mobilization of D3 was required and the location of the IDH was confirmed by needle aspiration. A Harmonic scalpel was utilised to incise the IDH longitudinally (Figure 2). Approximately 500 ml of blood clot was evacuated with a combination of suction and irrigation. The haematoma cavity was then explored with the 30 degree laparoscope to exclude a mucosal breach (Figure 3). A 14 F Kehr's "T" tube was placed in the cavity (Figure 4) and the seromuscular layer sutured closed with a 3-0 PDS continuous suture around this tube (Figure 5). A 10 F Jackson-Pratt drain was inserted in proximity to the drainage site.

Two days after surgery the NGT and Jackson-Pratt drain was removed and a free fluid diet commenced. The T tube was removed three days after surgery. The patient was discharged home on a normal diet four days after surgery. He had an uneventful recovery and no issues at follow-up.

## Discussion

Non-operative management of IDH is often successful. It represents the mainstream treatment of IDH unless active bleeding or bowel perforation is diagnosed and emergency laparotomy therefore required. In the majority of patients the gastric outlet obstruction secondary to IDH resolves after conservative measures including TPN and NGT treatment [6,8-10]. Only when these measures fail surgery is advocated.

The trend toward minimally invasive procedures has influenced the surgical management of IDH. Successful ultrasound or CT guided drainage has been reported IDH [11,12]. After 2 weeks from injury the haematoma is usually lysed and easier to aspirate [12]. Laparoscopic drainage of IDH has been described in the literature only twice. Banieghbal described a four port approach, similar to laparoscopic cholecystectomy, in an 11 year old child. An omental patch was applied on the serosa opening [13]. Maemura described an IDH in a 21 year old man following blunt abdominal trauma who required surgery due to evolving biliary obstruction [14]. The laparoscopic procedure was abandoned due the finding of a duodenal wall perforation, which required a laparotomy with formal repair and pyloric exclusion.

There are a number of points to detail about our laparoscopic approach. Firstly, the inframesocolic route allows a direct approach to the haematoma without need for a Kocher manoeuvre. The approach allows the entire clot to be evacuated and introduction of a laparoscope in the cavity allows limited assessment for mucosal lacerations. The T-tube assists decompression of the cavity should more bleeding occur or serum accumulate in the haematoma cavity. It also allows the development of a controlled fistula if a mucosal perforation has been missed at exploration of the cavity. We believe the technique is robust and simple and can be applied in most cases where conservative measures fail and facilitates early recovery and discharge from hospital.

## Conclusion

IDH is an uncommon injury after blunt abdominal trauma. Most patients can be treated conservatively with NGT decompression and TPN. When conservative management fails and drainage is required this can be safely achieved with a laparoscopic technique.

## Consent

Written informed consent was obtained from the patient for publication of this case report and accompanying images.

## Competing interests

The authors declare that they have no competing interests.

## Authors' contributions

GN prepared the manuscript and performed the literature review. CB formulated and assisted in the preparation of the manuscript. JG conceived and performed the technique described in this manuscript. All authors have read and approved the final manuscript.
